# Absence of multiplicative interactions between occupational lung carcinogens and tobacco smoking: a systematic review involving asbestos, crystalline silica and diesel engine exhaust emissions

**DOI:** 10.1186/s12889-017-4025-1

**Published:** 2017-02-02

**Authors:** Mohamad El Zoghbi, Pascale Salameh, Isabelle Stücker, Patrick Brochard, Fleur Delva, Aude Lacourt

**Affiliations:** 1 Univ. Bordeaux, Inserm, Bordeaux Population Health Research Center, team EPICENE, UMR 1219, Bordeaux, F-33000 France; 2 CHU de Bordeaux, Pole de sante publique, Service de médecine du travail et de pathologie professionnelle, Bordeaux, F-33000 France; 3 School of Pharmacy, Lebanese American University, Byblos, Lebanon; 4 Epidemiological & Clinical Laboratory Research, Faculty of Pharmacy, Lebanese University, Beirut, Lebanon; 5 Université Paris Saclay, University of Paris-Sud, UVSQ, CESP, INSERM, Villejuif, France

**Keywords:** Lung cancer, Interaction, Smoking, Occupational exposures

## Abstract

**Background:**

Tobacco smoking is the main cause of lung cancer, but it is not the sole causal factor. Significant proportions of workers are smokers and exposed to occupational lung carcinogens. This study aims to systematically review the statistical interaction between occupational lung carcinogens and tobacco smoking, in particular asbestos, crystalline silica and diesel engine exhaust emissions.

**Methods:**

Articles were identified using Scopus, PubMed, and Web of Science, and were limited to those published in English or French, without limitation of time. The reference list of selected studies was reviewed to identify other relevant papers. One reviewer selected the articles based on the inclusion and exclusion criteria. Two reviewers checked the eligibility of articles to be included in the systematic review. Data were extracted by one reviewer and revised by two other reviewers. Cohorts and case–control studies were analyzed separately. The risk of bias was evaluated for each study based on the outcome. The results of the interaction between the tobacco smoking and each carcinogen was evaluated and reported separately.

**Results:**

Fifteen original studies were included for asbestos-smoking interaction, seven for silica-smoking interaction and two for diesel-smoking interaction. The results suggested the absence of multiplicative interaction between the three occupational lung carcinogens and smoking. There is no enough evidence from the literature to conclude for the additive interaction. We believe there is a limited risk of publication bias as several studies reporting negative results were published.

**Conclusion:**

There are no multiplicative interactions between tobacco smoking and occupational lung carcinogens, in particular asbestos, crystalline silica and diesel engine exhaust emissions. Even though, specific programs should be developed and promoted to reduce concomitantly the exposure to occupational lung carcinogens and tobacco smoking.

**Electronic supplementary material:**

The online version of this article (doi:10.1186/s12889-017-4025-1) contains supplementary material, which is available to authorized users.

## Background

Lung cancer remains the leading cause of cancer deaths among males and females [1, 2]. In 1986, the International Agency for Research on Cancer (IARC) classified tobacco smoking as a lung carcinogen [3], it was identified as the main cause of lung cancer, and it was found to account for 80–90% of the cases [4, 5]. However tobacco smoking is not the sole causal factor of lung cancer. Indeed, lung cancer cases have been identified in non-smokers groups but exposed to different types of occupational exposures [6]. In the last update of IARC, almost 29 agents were classified as lung carcinogens with sufficient evidence in humans [7]. Many of them are found in occupational settings such as all forms of asbestos, crystalline silica and diesel engine exhaust emissions, which are among the top most frequent occupational exposures [8–11].

The rate of smoking is higher among blue-collar workers than white-collar workers [12]. Thus a significant proportion of workers are concomitantly exposed to occupational lung carcinogens and to tobacco smoking. This brings into light the importance of studying the statistical interactions between the occupational exposures and tobacco smoking.

In fact, the statistical interaction between the occupational exposure to asbestos and tobacco smoking was well studied through systematic reviews and meta-analyses, indicating the presence of a positive additive statistical interaction [13–15]. In the other hand, no systematic reviews were conducted to evaluate the statistical interactions between occupational exposure to crystalline silica and tobacco smoking or between diesel engine exhaust emissions and tobacco smoking.

Determine the nature of the statistical interaction between the occupational exposures and tobacco smoking is of high interest from a public health perspective, in particular to develop prevention programs. Therefore, we conducted a review on the interaction between the three most important occupational lung carcinogens, namely asbestos, crystalline silica and diesel engine exhaust emissions and tobacco smoking to define if the interaction nature is similar irrespective to the lung carcinogen, or if the interaction nature is specific for each carcinogen.

The objective of this study is to evaluate the statistical interactions between the occupational exposures and tobacco smoking, with limitation to the three principal lung carcinogens; asbestos, crystalline silica and diesel engine exhaust emissions, through a systematic review including cohort and case–control studies.

## Methods

This systematic review was reported based on the PRISMA checklist (2009) Additional file 1 and the PRISMA-P for developing review protocols (2015) [16, 17].

### Search strategy

Articles reviewed in this paper were identified using three bibliographic databases: PubMed, Scopus and Web of Science. The selected studies were limited to those published in English or French, without limitation of time. The most recent research was conducted in June 30, 2016.

For asbestos-tobacco, silica-tobacco and diesel-tobacco statistical interactions, all records of the three bibliographic databases were searched using the following key words respectively: ["asbestos" and "lung cancer" and "smoking"], ["silica" and "lung cancer" and "smoking"], and ["diesel" and "lung cancer" and "smoking"].

### Inclusion criteria

Studies were included in this review if they met the following criteria: human studies, studies published in peer-reviewed journals, cohort or case–control studies, studies evaluating the statistical interaction between tobacco smoking and one of the three studied occupational exposures on lung cancer, studies reporting the occupational exposure assessment, studies reporting the smoking behavior assessment, studies reporting the statistical analysis performed to assess the statistical interaction, and studies reporting the results of the statistical interaction and their statistical significance (*P*-value or CI 95%). For studies analyzing the same population, the most recently published article evaluating the statistical interaction that met all of the previous criteria was included.

### Exclusion criteria

In general, studies not meeting the inclusion criteria were excluded: clinical trials, in vitro studies, animal studies, cross-sectional studies, systematic reviews, meta-analyses, case reports and case series. Articles studying the statistical interactions between environmental exposures to asbestos, crystalline silica and diesel engine exhaust emissions and tobacco smoking on lung cancer were also excluded. Finally, articles that investigated the statistical interactions between asbestosis, silicosis, and smoking without taking into consideration asbestos and crystalline silica exposures were also excluded.

### Articles selection process

Records identified through the three bibliographic databases were checked for duplications. Duplicated records were removed, and the remaining records were screened to distinguish those that met the inclusion criterias. The screening phase was done in three steps: 1) selection of articles that studied the association between one of the three occupational exposures and lung cancer, 2) selection of the articles that studied the interaction based on the title or the abstract, and 3) for the remaining articles, the full-text was screened to select studies that evaluated the interaction between one of the three occupational exposures and smoking. The reference list of the selected articles was reviewed to identify other relevant articles. The full-text articles remained was assessed for eligibility to determine the final list of articles included in the qualitative synthesis.

### Data extraction

Data extraction was performed by one author (MZ), and reviewed by two other authors (FD and AL). The following data were extracted from each study included in the present review: first author, publication year, geographic area, study type (prospective cohort study, retrospective cohort study, nested case–control study, population-based case–control study, hospital-based case–control study), exposure type, industry type, total number of subjects (population and cases/cases and controls), the method to collect the occupational exposure and smoking status details, the definition of occupational exposure, the definition of smoking status, the outcome (lung cancer) classification, the methodology of the statistical interaction evaluation, and the results of the statistical interaction evaluation.

### Statistical interaction concepts

Rothman et al*.* stated that “the concept of interaction is that the effect of an exposure, compared with a reference unexposed group, may depend on the presence of one or more other factors”. In addition, they specified that the statistical interaction is potentially scale-dependent [18]. In epidemiologic studies, researchers examine the additive interaction or multiplicative interaction only for empirical reasons; and usually use the one that shows a better fit to the observations. In fact, statistical interactions are mostly evaluated on multiplicative scale, due to the statistical models used in the analyses (e.g. logistic regression), and that the models generate the multiplicative interaction result directly. If authors are interested in the evaluation of the statistical interaction, they should report results on additive and multiplicative scales [19]. The methods of the statistical interaction evaluation used in the original papers are described in more detail [see Additional file 2].

### Quality assessment and risk of bias

The Newcastle-Ottawa quality assessment scale (NOS) was used to assess the quality of the design and the conduction of the included studies at the outcome level [20, 21].

## Results

### Study selection

**Fig. 1:**
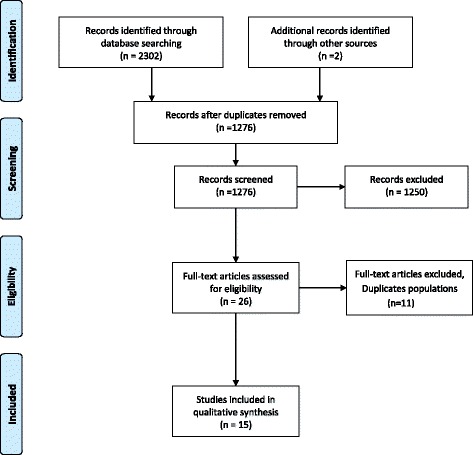
Study selection process for asbestos-smoking interaction

Using the methodology previously delineated, 2,302 articles were identified for the asbestos-smoking interaction: 1,061 from Scopus, 628 from PubMed, and 613 from Web of Science. In addition, two articles were added from the reference list of the selected articles. 1,028 articles were duplicated and excluded. From the remaining 1,276 articles, 1,250 papers were irrelevant; studies not meeting the inclusion criteria, or meeting the exclusion criteria. After screening phase, 26 full-text articles were assessed for eligibility; 11 articles were excluded because of duplicates population and 15 articles were retained including 6 cohorts, 1 case-cohort study, and 8 case–control studies (Fig. 1).

**Fig. 2:**
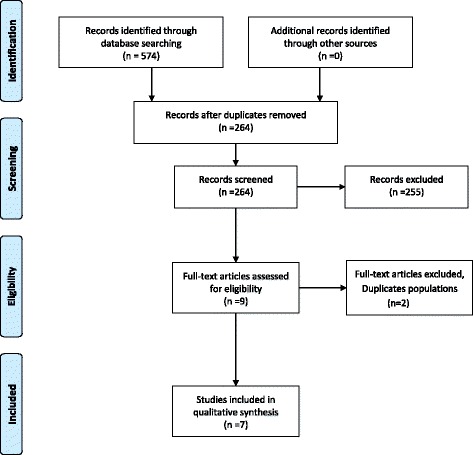
Study selection process for silica-smoking interaction

**Fig. 3:**
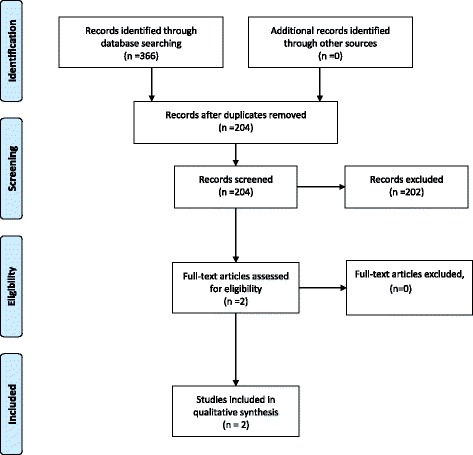
Study selection process for diesel-smoking interaction

The same methodology was used for silica-smoking and diesel-smoking interactions. In the end, seven articles were included for silica-smoking interaction involving one cohort, one nested case–control study, and five case–control studies (Fig. 2). For diesel-smoking interaction, only two articles were included involving one nested case–control study and one pooled case–control study (Fig. 3).

The different phases of the study selection for the interactions between the three different occupational exposures and smoking are presented using the PRISMA 2009 Additional file 1 flow diagrams [16].

### Occupational exposures and tobacco smoking interactions

#### Asbestos-smoking statistical interaction

The characteristics and the results of the six cohort studies and the case-cohort study evaluating asbestos-smoking statistical interaction are presented in Tables 1 and 2. Out of the seven studies, six studies assessed the multiplicative interaction; Multiplicative interaction was evaluated for the chrysotile workers of Qinghai mine in China [22], the crocidolite workers of Wittenoom mine in Australia [23], and the asbestos factory workers of East London [24]. The relative asbestos effect (RAE) with 95% confidence interval was calculated in the three studies, indicating the absence of a multiplicative interaction [22–24]. Additive and multiplicative interactions were evaluated for asbestos workers cohort in Great Britain [25]. Results showed that there is a positive additive interaction, but an absence of multiplicative interaction [25]. Additive and multiplicative interactions were also examined for the birth cohort of Quebec chrysotile miners and millers by calculating Rothman’s synergy index (*S*) and RAE, showing the absence of additive and multiplicative interactions [26]. The case-cohort study evaluated the interactions and showed the absence of additive and multiplicative interactions [27]. The additive interaction was assessed for a cohort of Chinese male asbestos plant workers by calculating *S*. The value of *S* was not significantly greater than one indicating the absence of additive interaction [28].

**Table 1:** Description of cohort studies included in the systematic review for the asbestos-smoking interaction

Author (Year)	Study design	Geographic area	Industry type	Fiber type	Outcome identification	Asbestos				Smoking			NOS
						Data collection	Exposure Identification	Non exposed	Exposed	Data collection	Non Smoker	Smoker	
Berry (1985) [24]	Prospective	East London (UK)	Asbestos factory	Undetermined	Register	Employment record	Expert	Light and moderate	Severe	Questionnaire	Never	Ever	6
Liddell (2002) [26]	Prospective	Quebec (Canada)	Mining and Milling	Chrysotile	Death certificate	Employment record	Measures	<30 mpcf.y	≥30 mpcf.y	Questionnaire	Never	Ever	7
Reid (2006) [23]	Prospective	Wittenoom (Australia)	Mining and Milling	Crocidolite	Register	-Questionnaire -Employment record	Measures	Low	High	Questionnaire	Never and former >20 Y	Current and former <20 Y	8
Frost (2011) [25]	Prospective	Great Britain	Different types	Undetermined	Register	Questionnaire	Expert	<10 Y	≥30 Y	Questionnaire	Never	Current	8
Wang (2012) [22]	Prospective	Qinghai (China)	Mining and Milling	Chrysotile	Death certificate	Employment record	Measures	Non miners and millers	Miners and millers	Interview	Never	Ever	6
Wang (2012) [28]	Prospective	China	Asbestos factory	Chrysotile	Death certificate	-Employment record -Questionnaire	Measures	Electronics factory	Asbestos cohort	Questionnaire	Never	Ever	7
Offermans (2014) [27]	Case-cohort	Netherlands	Undetermined	Undetermined	Register	Questionnaire	Matrix	Never	Ever	Questionnaire	Never	Current	7

*NOS* the Newcastle-Ottawa quality assessment Scale, *S* current smokers, *mpcf.y* million particles per cubic foot x years, *Y* years

**Table 2:** Results of cohort studies included in the systematic review for the asbestos-smoking interaction

Author (Year)	Subjects	Cases (sub-cohort)	Follow-up	NS/Exposed	S/Exposed	Interaction Estimation		Interpretation	
						Additive	Multiplicative	Additive	Multiplicative
Berry (1985) [24]	M: 1250 F: 420	M: 64 F: 15	1971–1980	RR = 7.3	RR = 2.4	NA	RAE = 3.0 (0.8-7.5)	NA	No
Liddell (2002) [26]	M: 7279	M: 533	1904–1992	SMR = 0.62	SMR = 1.71	S = 1.1 (0.73-1.41)	RAE = 1.69 (0.87-3.28)	No	No
Reid (2006) [23]	M: 2550 F: 183	M: 132 F: 6	1979–2002	OR = 2.02 (0.61–6.72)	OR = 1.64 (1.124–2.37)	NA	RAE_m_ = 1.59 (0.12-20.50)	NA	No
Frost (2011) [25]	M: 93966	M: 1768	1971–2005	RR = 1.6 (0.6–4.2)	RR = 26.2 (13.0–53.1)	S = 1.4 (1.2-1.6)	V = 0.9 (0.3-2.4)	Positive	No
Wang (2012) [22]	M: 1539	M: 50	1981–2006	SMR = 1.79 (0.49-6.51)	SMR = 5.45 (4.11-7.22)	NA	RAE = 0.52 (0.07-1.11)	NA	No
Wang (2012) [28]	M: 577	M: 53	1972–2008	HR = 7.52 (0.90-62.79)	HR = 17.35 (2.38-126.57)	S = 1.41 (0.73-3.99)	NA	No	NA
Offermans (2014) [27]	M: 2324	M: 2107	1986–2003	HR = 1.79 (1.04–3.08)	HR = 10.21 (7.26–14.35)	RERI = 1.94 (0.13-4.89)	IT P = 0.50	No	No

*M* Males, *F* Females, *S* smokers, *NS* non-smokers, *S* Synergy Index, *RERI* Relative Excess Risk due to Interaction*, V* Multiplicativity Index, *RAE* Relative Asbestos Effect, *RAE*_*m*_ the Modified Relative Asbestos Effect

The characteristics and the results of the eight case–control studies evaluating asbestos-smoking statistical interaction are presented in Tables 3 and 4. Seven of those studies did not reveal any multiplicative interaction when they were assessed [6, 29–34]. One case–control study, conducted in Sweden, evaluated the additive and multiplicative interactions and showed the absence of an additive interaction and the presence of a negative multiplicative interaction [35].

**Table 3:** Description of case–control studies included in the systematic review for the asbestos-smoking interaction

Author (Year)	Study design	Geographic area	Subjects identification		Asbestos					Smoking		NOS
			Case	Control	Data collection	Exposure Identification	Non exposed	Exposed	Data collection	Non Smoker	Smoker	
Martischnig (1977) [29]	Hospital-based	United Kingdom	Hospital	Hospital	Questionnaire	Expert	No occupational history	Occupational history	Questionnaire	0-14 C/D	>14 C/D	7
Blot (1978) [30]	Hospital-based	Coastal Georgia (USA)	-Hospital -Death certificate	Hospital	Interview	Auto-declaration	Not shipbuilder	Shipbuilder	Interview	<10 C/D	≥10 C/D	6
Jöckel (1998) [31]	Hospital-based	Germany	Hospital	Register	-Questionnaire -Interview	Matrix	Never	Exposed >5280 h	-Questionnaire -Interview	Never and < 27.1 P.Y	≥27.1 P.Y	7
Gustavsson (2002) [35]	Population-based	Sweden	Register	Register	Self-reported questionnaire	Industrial hygienist	Never	≥1.0 f-years	Self-reported questionnaire	Never	Current	7
Carel (2007) [32]	Hospital-based	Europe	Hospital	-Hospital -Register	Questionnaire	Expert	Never	Exposed > 1 Y	Questionnaire	Never	Ever	8
De Matteis (2012) [6]	Population-based	Italy	Hospital	Population databases	Questionnaire	Matrix	Never	Ever	Questionnaire	Never	-Former -Current	6
Villeneuve (2012) [33]	Population-based	Canada	Register	General population	Self-reported questionnaire	Industrial hygienist	Never	Medium or high	Self-reported questionnaire	<10 P.Y	≥10 P.Y	7
Lacourt (2015) [34]	Pooled	Montreal (Canada)	Hospital	Population (electoral lists)	Questionnaire	-Chemist -Industrial hygienist	Never	Ever	Questionnaire	Never-low	Medium-heavy	7

*NOS* the Newcastle-Ottawa quality assessment Scale, *S* current smokers, *Y* years, *C/D* cigarettes per day, *P.Y* Pack.Year

**Table 4:** Results of case–control studies included in the systematic review for the asbestos-smoking interaction

Author (Year)	Cases	Controls	Recruitment period	NS /Exposed	S/Exposed	Interaction Estimation		Interpretation	
						Additive	Multiplicative	Additive	Multiplicative
Martischnig (1977) [29]	M: 201	M: 201	1972–1973	RR = 1.08	≥25 C/D: RR = 3.26	NA	No heterogeneity of RRs X^2^ = 2.89; DF =2	NA	No
Blot (1978) [30]	M: 458	M: 553	1970–1976	RR = 1.3	RR = 1.7 RR = 2.4	NA	No heterogeneity of RRs *P* > 0.10	NA	No
Jöckel (1998) [31]	M: 839 F: 165	M: 839 F: 165	1988-1993	OR = 1.1	OR = 6.5 OR = 18	NA	IT *P* = 0.73	NA	No
Gustavsson (2002) [35]	M: 1038	M: 2359	1985–1990	RR = 4.2 (1.6-11.1)	RR = 28.6 (19.9-48.3)	S = 1.15 (0.77-1.72)	IT OR = 0.31 (0.11-0.86)	No	Negative
Carel (2007) [32]	M: 2205	M: 2305	1998–2002	Not shown	Not shown	NA	No modification effect CEE: *P* = 0.37 UK *P* = 0.62.	NA	No
De Matteis (2012) [6]	M: 1537	M: 1617	2002–2005	OR = 2.47 (1.15-5.31)	OR = 49.54 (28.18-87.08)	NA	LRT *P* = 0.19	NA	No
Villeneuve (2012) [33]	M: 1681	M: 2053	1994–1997	OR = 2.20 (0.42-11.41)	OR = 38.59 (10.78-138.08)	NA	IT *P* = 0.77	NA	No
Lacourt (2015) [34]	M: 414	M: 321	St.1: 1979–1986 St.2: 1996–1998	OR = 1.2 (0.7–2.2)	OR = 1.1 (0.8–1.6)	NA	IT *P* = 0.68	NA	No

*M* Males, *F* Females, *S* Smokers, *NS* Non-Smokers, *CEE* Central and Eastern Europe, *St* Study, *S* Synergy Index, *LRT* likelihood ratio test, *IT* interaction term

#### Silica-smoking statistical interaction

The characteristics and the results of the studies evaluating silica-smoking statistical interaction are presented in Tables 5 and 6. One cohort and one nested case–control study were reviewed and included. The cohort study, published in 2013, evaluated the additive and the multiplicative silica-smoking statistical interaction. The results of this study indicated the absence of additive and multiplicative interactions [36]. The nested case–control study examined the multiplicative interaction by adding an interaction term of crystalline silica exposure and smoking to the logistic regression, and showing the absence of a multiplicative interaction [37].

**Table 5:** Description of silica-smoking and diesel-smoking interaction studies included in the systematic review

Author (Year)	Study design	Geographic area	Industry type	Outcome identification	Silica				Smoking			NOS
					Data collection	Exposure Identification	Non exposed	Exposed	Data collection	Non Smoker	Smoker	
Silica-smoking interaction studies												
Liu (2013) [36]	Prospective cohort	China	Metal mines and pottery factories	-Hospital record -Death certificate	Employment record	Matrix	Never	Ever	Interview	Never	Ever	8
Fu (1994) [37]	Nested case–control	Guangxi province (China)	Tin miners	Death Certificate	-Employment record -Questionnaire	Expert	Never	YUED	Questionnaire	Never	P.Y	7
Cassidy (2007) [38]	Multicenter hospital-based case–control	Europe	Undetermined	Hospital	Questionnaire	Industrial hygienist	Never	Ever	Questionnaire	Never	-Former -Current	7
De Matteis (2012) [6]	Population-based case–control	Italy	Undetermined	Hospital	-Questionnaire -Interview	Matrix	Never	Ever	-Questionnaire -Interview	Never	-Former -Current	6
Kachuri (2014) [39]	Population-based case–control	Canada	Undetermined	Register	Self-reported questionnaire	Industrial hygienist	Never	≥30 Y	Self-reported questionnaire	<10 P.Y	≥40 P.Y	8
Consonni (2015) [40]	Pooled case–control	Europe, Canada, Hong Kong and New Zealand	Bricklayers	-Register -Hospital	Questionnaire	Matrix	Never bricklayers	Ever bricklayers	Questionnaire	Never	Ever	8
Lacourt (2015) [34]	Pooled case–control	Montreal (Canada)	Construction	Hospital	Questionnaire	Expert	Never	Substantial	Questionnaire	Never-low	Medium-heavy	7
Diesel-smoking interaction studies												
Silverman (2012) [41]	Nested case–control	USA	Non-metal mining facilities	-Register -Death certificate	Computer-assisted telephone interview	Measures	Never	Tertiles	Computer-assisted telephone interview	Never	≥2 P.D	8
Pintos (2012) [42]	Pooled case–control	Canada	Wide range of occupations and industries	Incident case	Questionnaire	-Chemist -Industrial hygienist	Never	Substantial	Questionnaire	Never-low (0–15 P.Y)	Medium-heavy (>15 P.Y)	7

*NOS* the Newcastle-Ottawa quality assessment Scale, *S* current smokers, *Y* years, *YUED* Years of Underground Exposure to Dust, *P.Y* Pack.Year

**Table 6:** Results of silica-smoking and diesel-smoking interaction studies included in the systematic review

Author (Year)	Subjects (cases)	Cases (controls)	Period^a^	NS/Exposed	S/Exposed	Interaction Estimation		Interpretation	
						Additive	Multiplicative	Additive	Multiplicative
Silica-smoking interaction studies									
Liu (2013) [36]	34018	546	1960–2003	HR = 1.10 (0.68-1.78)	HR = 3.83 (2.48-5.90)	RERI = 0.98 (0.23-1.74)	IT *P* = 0.25	No	No
Fu (1994) [37]	M: 79	M: 188	1973–1989	NA	NA	NA	IT *P* = 0.57	NA	No
Cassidy (2007) [38]	M: 2197 F: 655	M: 2295 F: 809	1998–2002	OR = 1.41 (0.79 -2.49)	OR = 1.41 (1.07-1.87)	NA	Test for Heterogeneity *P* = 0.37	NA	No
De Matteis (2012) [6]	M: 1537	M: 1617	2002–2005	OR = 1.41 (0.51-3.91)	OR = 44.98 (27.15-74.52)	NA	LRT *P* = 0.94	NA	No
Kachuri (2014) [39]	M: 1681	M: 2053	1994–1997	OR = 0.63 (0.26-1.52)	OR = 42.53 (23.54-76.83)	S = 2.38 (1.35-4.21)	V = 3.59 (1.51-8.49)	Positive	Positive
Consonni (2015) [40]	M: 15608	M: 18531	1985–2010	OR = 1.18	OR = 18.5	RERI = 6.80 (4.36-9.62)	IT *P* = 0.28	Positive	No
Lacourt (2015) [34]	M: 241	M: 196	St.1: 1979–1986 St.2: 1996–1998	OR = 3.1 (1.0–9.6)	OR = 1.4 (0.7–2.7)	NA	IT *P* = 0.02	NA	Negative
Diesel-smoking interaction studies									
Silverman (2012) [41]	M: 198	M: 562 Ma	1947–1977	OR = 7.30 (1.46- 36.57)	OR = 17.38 (3.48-86.73)	NA	IT *P* = 0.086	NA	No
Pintos (2012) [42]	St. I: M: 857 St. II: M: 736	St. I: M: 533 St. II: M: 894	St. I: 1979–1986 St. II: 1996–2001	OR = 2.29 (1.1-4.6)	OR = 9.84 (6.4-15.1)	NA	IT OR = 1.15 (0.5-2.7)	NA	No

*M* Males, *F* Females, *S* Smokers, *NS* Non-Smokers, *NA* Not Applicable, *S* Synergy Index, *V* Multiplicativity Index, *RERI* Relative Excess Risk due to Interaction, *LRT* likelihood ratio test, *IT* interaction term

^a^: Follow-up or recruitment period

Five case–control studies were reviewed in this study to assess the silica-smoking statistical interaction. Two studies, one conducted in several centers in Europe and the other in Italy, showed that there is no multiplicative interaction [6, 38]. A study published in 2015, evaluated the multiplicative interaction between the exposure of construction workers to crystalline silica and smoking. The study showed a negative multiplicative interaction; the effect of occupational exposure to crystalline silica was higher for non/light smokers than for medium/heavy smokers [34]. A population-based case–control study in eight Canadian provinces showed positive additive and positive multiplicative interactions [39]. Another pooled case–control study (SYNERGY study) showed positive additive interaction, but no multiplicative interaction [40].

#### Diesel-smoking statistical interaction

Only two articles assessed the diesel-smoking statistical interaction were included in our review (Table 5). These two studies presented a nested case–control study of the workers of eight non-metal mining facilities in United States [41] and a pooled case control study conducted in Montreal (Canada) [42]. The results of these two studies (Table 6) showed the absence of a multiplicative interaction [41, 42].

## Discussion

Overall, this review suggests the absence of a multiplicative statistical interaction between the three most frequent occupational lung carcinogens, asbestos, crystalline silica and diesel engine exhaust emissions and tobacco smoking. On the other side, there is no enough evidence from the literature to conclude on the additive statistical interaction.

### Asbestos-smoking statistical interaction

Four meta-analyses were conducted to evaluate the asbestos-smoking statistical interaction; one demonstrated a negative multiplicative interaction [43], and three suggested the presence of a positive additive interaction [13–15]. The most recent systematic review published in 2015 indicated the presence of a positive additive interaction and the absence of multiplicative interaction [15]. While we agreed about the absence of a multiplicative interaction, from this systematic review, the presence of a positive additive interaction is less clear. Indeed, out of the five original studies included in this review, only one showed a significant positive additive interaction. This discordance is mainly attributable to selection criteria of original studies. While in the most recent meta-analysis, authors included all studies from which they could assess statistical interaction from odds ratios or relative risks reported in the original studies without any notion of statistical significance [15], in the present systematic review, we add more stringent inclusion criteria. Indeed, we only included and evaluated studies that reported both the interaction results on a specified scale (multiplicative or additive) and the significance of the results, either in terms of confidence intervals or *p*-value,. However, conclusions from our study are based on a systematic review of the literature and we did not perform a meta-analysis since it was not the primary aim of this study to focus exclusively on the asbestos-smoking statistical interaction. Instead, the present study aimed at assessing the statistical interactions between the most frequent occupational lung carcinogen and tobacco-smoking. Despite the recent publication of a meta-analysis assessing the asbestos-smoking statistical interaction, performing a new one using more stringent inclusion criteria for studies should be considered.

### Silica-smoking and diesel smoking statistical interaction

Similarly to asbestos-smoking statistical interaction, for both silica-smoking as well as diesel-smoking statistical interaction, the absence of a multiplicative statistical interaction seems to be consensual. Regarding additive interaction, for both silica-smoking and diesel-smoking statistical interaction, it is impossible to conclude on the presence of a statistical interaction on the additive scale. Indeed, for silica-smoking interactions, it is impossible to conclude due to discrepancies between original studies whereas for diesel-smoking interaction, no studies included in the present systematic review have addressed this issue.

### Methodological points in original studies

The inconsistency of the statistical interaction results between original studies may come from methodological differences in each study. Every study has limitations that could be the source of opposite results on the interaction evaluation. In the studies that were included, occupational histories and smoking details were collected using employment records or questionnaires. The reliability of the data may have been affected by the quality of the documentation in the records and by the recall bias from the questionnaires used to collect retrospective data. Although the data collection could be complete and accurate, the methods used to identify and assess occupational exposures may also have been a source of bias. For example; the utilization of a job-exposure matrix (JEM) could introduce non-differential misclassifications leading to a large number of false-positives and false-negatives. In consequence, there is a risk of underestimated risks that could affect the evaluation of the interaction [44, 45].

When evaluating interactions, the method and the scale used to examine the interaction should be reported to avoid confusion and ambiguity and facilitate the comparison between studies [46]. In fact, the best approach is to evaluate the statistical interaction on both additive and multiplicative scales [19]. The additive interaction is generally evaluated by using the difference of risk differences known as interaction contrast, while risk ratios are used to evaluate the multiplicative interactions. In cohort studies, risks and risk ratios can be easily generated, but in the case–control studies only the odds ratios can be estimated. Using odds ratios instead of risk ratios to evaluate the additive or the multiplicative interaction could mistakenly show the presence of a positive interaction, even if the outcome is rare [47, 48]. The majority of the reviewed case–control studies evaluated exclusively the multiplicative interaction by testing the significance of the interaction term introduced into the regression model. However, while rarely used, some authors have proposed various measures to assess the additive interaction from case–control data using logistic regression models [49–52]. Additionally, discrepancy between studies may be explained by the measures used to assess the statistical additive interaction as each measure has its own interpretation. Indeed, Rothman et *al.* and Kalilani et *al.* suggested to use simultaneously three measures of interaction to evaluate the additive interaction: the attributable proportion due to interaction (AP), the relative excess risk due to interaction (RERI), and the Rothman’s synergy index (*S*) [53, 54]. Although, the attributable proportion due to interaction (AP) is the most robust measure to evaluate the additive interaction when the odds ratios are used instead of the risk ratios in the equation [54]. Because of its more intuitive interpretation, the Rothman’s synergy index (*S*) [55] was used in the majority of the included studies to evaluate the additive statistical interaction even when odds ratios were used instead of risk ratios. Indeed, both S and AP measure interaction as departure from additivity but only S is suitable under a negative additive interaction assumption. Specific measures of interaction have been proposed to assess the statistical multiplicative interaction between asbestos exposure and tobacco smoking. The RAE was proposed to evaluate the asbestos-smoking multiplicative interaction in cohort studies [24]. However it was shown that the RAE tended to be underestimated in studies with low level of asbestos exposure. Thus, a modified version of the RAE (RAE_m_) have been proposed to assess the asbestos-smoking multiplicative interaction in studies with low asbestos exposure level [43].

In many of the reviewed articles, the conclusion regarding the statistical interaction was not always consistent with the results of our evaluation; authors suggested the presence of a positive interaction without evaluating the statistically significance of the measure, or conclude on both scales although the interaction was evaluated on one scale only. The same findings was discussed by Liddell (2001); authors continue to suggest the presence of a positive multiplicative asbestos-smoking interaction without enough or strong evidence from their results or from the literature [56].

In the current review, our conclusions are based on strong evidence, as the majority of the reviewed studies conclude the absence of the multiplicative interaction. In addition, all precautions were taken to avoid missing papers; three different bibliographic databases were used and each reference list of all included studies was reviewed. Finally, we believe that the publication bias is limited as several papers with negative results were published.

### Public health implications

Statistical interaction (whatever the model, multiplicative or additive) between two risk factors increases cancer risk compared to risk related to each factors acting independently.

Two main impacts can be considered from a public health point of view. First, regarding primary prevention, reducing exposure to those two risk factors will induce a greater benefice (number of avoided incident cases) if there is a significant interaction between those two factors. Secondly, regarding targeted screening program (screening proposed to a selected population according to a specific risk threshold), the existence of an interaction will decrease the level of exposure of those two factors corresponding to the defined risk threshold. The same argument could be applied to individual imputability used in compensation system. Therefore, the knowledge of a statistical interaction between two risk factors is crucial and the knowledge of the interaction scale (i.e. multiplicative or additive) is important to conduct risk assessment and risk management.

Besides, in the light of the current knowledge, the statistical interaction between two factors do not allow to infer strong hypothesis about biological mechanisms

## Conclusions

To our knowledge, this is the first systematic review conducted to evaluate the statistical interactions between occupational exposures to crystalline silica and diesel engine exhaust emissions and tobacco smoking. In general, there is no multiplicative interaction between the three most frequent occupational lung carcinogens and the tobacco smoking. Evidence found in the literature cannot be considered sufficient to conclude on the additive scale. To minimize the risk of lung cancer among workers, specific programs should be developed and promoted to reduce concomitantly the exposure to occupational lung carcinogens and tobacco smoking.

## Additional files

Additional file 1. PRISMA 2009 Checklist. (DOC 62 kb)
Additional file 2. Statistical Interaction Evaluation. (DOCX 72 kb)

## Abbreviations

AP: the attributable proportion due to interaction
IARC: International agency for research on cancer
NOS: The Newcastle-Ottawa quality assessment scale
RAE: the relative asbestos effect
RAE_m_: the modified version of the relative asbestos effect
RERI: Relative excess risk due to interaction
S: Rothman’s synergy index
