# Supplementary material for: Absence of multiplicative interactions between occupational lung carcinogens and tobacco smoking: a systematic review involving asbestos, crystalline silica and diesel engine exhaust emissions
Source: BMC Public Health. 2017 Feb 2;17:156. doi: 10.1186/s12889-017-4025-1 (PMC5288859; doi:10.1186/s12889-017-4025-1)
Supplement: Additional file 2. — Statistical Interaction Evaluation. (DOCX 72 kb) [file 12889_2017_4025_MOESM2_ESM.docx]

## Additional file 2

## Statistical Interaction Evaluation

The statistical interaction is equivalent to the concept of effect-measure modification or heterogeneity of effect; this could occur on both additive and multiplicative scales. There is effect-measure modification, or statistical interaction, on the difference scale (additive scale), when the risk differences are heterogeneous across categories of the other factor. Otherwise, there is effect-measure modification, or statistical interaction, on the risk ratio scale for the effect (multiplicative scale), when the risk ratios for one factor are heterogeneous across categories of the other factor. Rothman et *al.* demonstrated that if both factors have effects, and there is no modification (heterogeneity) of the risk differences for one factor by the other factor, there has to be modification of the risk ratios, and vice versa.[[1](#_ENREF_1)] In different studies, authors used Rothman demonstration to conclude, for example, there is a multiplicative interaction, when the additive interaction is not evident. For the purpose of this review, we have determined the nature of the statistical interaction based exclusively on the scale(s) used in the study.

In cohort studies, risks and risk ratios can be easily generated and used to evaluate the additive and the multiplicative interactions, respectively. The additive interaction is generally evaluated by using the difference of risk differences known as Interaction Contrast (IC), while risk ratios are used to evaluate the multiplicative interactions. Unfortunately, in case-control and cross-sectional studies, the risks cannot be estimated and the risk ratios cannot be calculated, but odds ratios are calculated and used as approximately equal to risk ratios when the outcome (e.g. lung cancer) is rare. Therefore, the multiplicative interaction is only evaluated [[2](#_ENREF_2), [3](#_ENREF_3)]**.**

However, Rothman et al proposed three alternative measures based on risk ratios to evaluate the additive interaction: 1) the Rothman’s synergy index (S; equation 1), 2) the Relative Excess Risk due to Interaction (RERI; equation 2), also known as the Interaction Contrast Ratio [[4-6](#_ENREF_4)] ICR) and 3) Attributable Proportion due to interaction (AP; equation 3). [[7](#_ENREF_7)] In the following equations, RR00 is the relative risk of persons not exposed to either factor (reference group), RR10 is the relative risk due to smoking, RR01 is the relative risk due to occupational exposure, and RR11 is the relative risk due to both factors.

*S* equation is:

$S=\frac{R11-R00}{\left( R10-R00 \right)+\left( R01-R00 \right)}=\frac{RR11-1}{\left( RR10-1 \right)+(RR01-1)}$ Equation 1

*S* is the excess risk from both factors when there is interaction relative to the excess risks of each factor in the absence of the other without interaction. A value of *S* significantly above (or below) one indicates the presence of an additive interaction [[4](#_ENREF_4), [5](#_ENREF_5), [8](#_ENREF_8)].

RERI equation is:

$RERI=ICR= \frac{IC}{R00}= \frac{R11-R10-R01+R00}{R00}=RR11-RR10-RR01+1$ Equation 2

RERI is the excess risk due to interaction relative to the risk without exposure. A value of RERI significantly above or below zero indicates an additive interaction [[4-6](#_ENREF_4)].

AP equation is:

$AP=\frac{R11-R10-R01+R00}{R11}=\frac{RR11-RR10-RR01+1}{RR11}=\frac{RERI}{RR11}$ Equation 3

AP is the attributable proportion of disease due to interaction among persons with both exposures. A value of AP that is significantly above zero indicates an additive interaction [[4](#_ENREF_4)]. After the development of these alternative measures, risk ratios were widely used to evaluate additive and multiplicative interactions.

In case-control studies, the odds ratios are approximately equal to risk ratios when the outcome is rare, so authors use them directly to evaluate the additive interaction by substituting risk ratios (RRs) by odds ratios (ORs) in equations. Thus, the three measures of additive interaction based on RRs are properly estimated, only when ORs are good estimates of RRs. In addition, the selection of the measure to evaluate the interaction on a scale is important; AP is the best measure to be used when there is a positive additive interaction, while RERI (ICR) and S are the best measures when there is a negative additive interaction [[3](#_ENREF_3)].

To evaluate the multiplicative interaction, the multiplicativity index (*V*) was developed and used in cohort, case-control and cross-sectional studies. [[9](#_ENREF_9)] The equation of the multiplicativity index (*V*) is:

$V=\frac{RR11*RR00}{RR10*RR01}$ Equation 4

A value of *V* which is statistically greater (or less) than one, indicates a multiplicative interaction [[9](#_ENREF_9), [10](#_ENREF_10)].

In addition to the multiplicativity index, the Relative Asbestos Effect (RAE) was used to evaluate the multiplicative interaction between smoking and asbestos. The RAE is the effect of smoking on the asbestos effect expressed as the ratio of the risk ratio due to asbestos exposure in non-smokers (RR01) to that in smokers (RR11). This measure is used to examine the heterogeneity of asbestos effect across categories of smoking status, to determine if there is effect-measure modification. If the asbestos effect is the same for non-smokers as for smokers, then the RAE is equal to one, whereas a value greater than one indicates that the effect of asbestos is greater in non-smokers than in smokers (negative multiplicative interaction), and vice versa [[11](#_ENREF_11)]. The equation of RAE is:

$RAE=\frac{RR01}{RR11}$ Equation 5

In 2004, Berry and Liddell proposed a modified measure of Relative Asbestos Effect (RAE_m_), defined as the ratio of the excess risk ratio due to asbestos exposure in non-smokers (RR01 – 1) to that in smokers (RR11 – 1). This modified measure was proposed because the original measure (RAE) cannot be generalized as a measure of the interactive effect for the low levels of asbestos exposure. The value of RAE_m_ would be higher as that of RAE. A value of RAE or RAE_m_ that is statistically greater (or less) than one, indicates a multiplicative interaction [[12](#_ENREF_12)].

The equation of RAE_m_ is:

$\mathrm{RAE}m=\frac{RR01-1}{RR11-1}$ Equation 6

In addition to these measures, logistic and Cox regressions were used to evaluate the multiplicative interaction, by adding an interaction term of occupational exposure and smoking to the model. A significant OR (95% CI) or a significant likelihood ratio test indicates a multiplicative interaction [[13-15](#_ENREF_13)].

**References**

1. Rothman JK, Greenland S, Lash LT: **Modern epidemiology**: Lippincott Williams & Wilkins; 2008.

2. Kupper LL, Hogan DM: **Interaction in epidemiologic studies**. *American journal of epidemiology* 1978, **108**:447-453.

3. Kalilani L, Atashili J: **Measuring additive interaction using odds ratios**. *Epidemiologic perspectives & innovations: EP+I* 2006, **3**.

4. Assmann FS, Hosmer WD, Lemeshow S, Mundt AK: **Confidence intervals for measures of interaction**. *Epidemiology (Cambridge, Mass)* 1996, **7**:286-290.

5. Hosmer WD, Lemeshow S: **Confidence interval estimation of interaction**. *Epidemiology (Cambridge, Mass)* 1992, **3**:452-456.

6. Richardson BD, Kaufman SJ: **Estimation of the relative excess risk due to interaction and associated confidence bounds**. *American journal of epidemiology* 2009, **169**:756-760.

7. Rothman JK: **Modern Epidemiology**; 1986.

8. Rothman JK: **Synergy and antagonism in cause-effect relationships**. *American journal of epidemiology* 1974, **99**:385-388.

9. Lee PN: **Relation between exposure to asbestos and smoking jointly and the risk of lung cancer**. *Occupational and environmental medicine* 2001, **58**(3):145-153.

10. Frost G, Darnton A, Harding AH: **The effect of smoking on the risk of lung cancer mortality for asbestos workers in Great Britain (1971-2005)**. *The Annals of occupational hygiene* 2011, **55**(3):239-247.

11. Berry G, Newhouse ML, Antonis P: **Combined effect of asbestos and smoking on mortality from lung cancer and mesothelioma in factory workers**. *British journal of industrial medicine* 1985, **42**(1):12-18.

12. Berry G, Liddell FD: **The interaction of asbestos and smoking in lung cancer: a modified measure of effect**. *The Annals of occupational hygiene* 2004, **48**(5):459-462.

13. De Matteis S, Consonni D, Lubin JH, Tucker M, Peters S, Vermeulen R, Kromhout H, Bertazzi PA, Caporaso NE, Pesatori AC *et al*: **Impact of occupational carcinogens on lung cancer risk in a general population**. *International journal of epidemiology* 2012, **41**(3):711-721.

14. Gustafson P, Kazi RAM, Levy RA: **Extending logistic regression to model diffuse interactions**. *Statistics in medicine* 2005, **24**:2089-2104.

15. Consonni D, De Matteis S, Pesatori AC, Cattaneo A, Cavallo DM, Lubin JH, Tucker M, Bertazzi PA, Caporaso NE, Wacholder S *et al*: **Increased lung cancer risk among bricklayers in an Italian population-based case-control study**. *American journal of industrial medicine* 2012, **55**(5):423-428.
